# Overexpression of an ALS-associated FUS mutation in *C. elegans* disrupts NMJ morphology and leads to defective neuromuscular transmission

**DOI:** 10.1242/bio.055129

**Published:** 2020-12-07

**Authors:** Sebastian M. Markert, Michael Skoruppa, Bin Yu, Ben Mulcahy, Mei Zhen, Shangbang Gao, Michael Sendtner, Christian Stigloher

**Affiliations:** 1 University of Würzburg, Biocenter, Imaging Core Facility, Am Hubland, Würzburg 97074, Germany; 2 University Hospital Würzburg, Institute of Clinical Neurobiology, Versbacherstraße 5, 97080 Würzburg, Germany; 3 Huazhong University of Science and Technology, Key Laboratory of Molecular Biophysics of the Ministry of Education, College of Life Science and Technology, Wuhan 430074, China; 4 Lunenfeld-Tanenbaum Research Institute, Mount Sinai Hospital, 600 University Avenue, Toronto, Ontario M5G 1X5, Canada; 5 University of Toronto, Department of Molecular Genetics, Physiology and Institute of Medical Science, 1 King's College Circle, Toronto, Ontario M5S 1A8, Canada

**Keywords:** *C. elegans*, Fused in sarcoma, Amyotrophic lateral sclerosis, Super-resolution array tomography, Electron tomography, Neuromuscular junction

## Abstract

The amyotrophic lateral sclerosis (ALS) neurodegenerative disorder has been associated with multiple genetic lesions, including mutations in the gene for fused in sarcoma (FUS), a nuclear-localized RNA/DNA-binding protein. Neuronal expression of the pathological form of FUS proteins in *Caenorhabditis elegans* results in mislocalization and aggregation of FUS in the cytoplasm, and leads to impairment of motility. However, the mechanisms by which the mutant FUS disrupts neuronal health and function remain unclear. Here we investigated the impact of ALS-associated FUS on motor neuron health using correlative light and electron microscopy, electron tomography, and electrophysiology. We show that ectopic expression of wild-type or ALS-associated human FUS impairs synaptic vesicle docking at neuromuscular junctions. ALS-associated FUS led to the emergence of a population of large, electron-dense, and filament-filled endosomes. Electrophysiological recording revealed reduced transmission from motor neurons to muscles. Together, these results suggest a pathological effect of ALS-causing FUS at synaptic structure and function organization.

This article has an associated First Person interview with the first author of the paper.

## Abstract (precis)

**Summary:** Neuronal expression of an ALS-associated mutation in a DNA/RNA-binding protein disrupts the organization and function of the *C. elegans* neuromuscular junction.

## INTRODUCTION

Amyotrophic lateral sclerosis (ALS) is a severe disease of the locomotor system where motor neurons progressively degenerate and muscles atrophy (Hardiman et al., 2017). ALS is hypothesized to be a synaptopathy (Fogarty, 2019), because the disease usually starts with dysfunction and degeneration of synapses, before axons and dendrites become dystrophic and the neurons undergo cell death (Chou, 1992). Most cases of ALS (∼90%) are considered ‘spontaneous’ – due to a combination of *de novo* genetic and environmental factors. The remaining ∼10% of ALS cases are hereditary and are caused by defined mutations in different genes. A number of cellular defects have been implicated in the pathophysiology of ALS, including defective endosomal and receptor trafficking, as well as changes in autophagy and axonal transport (Burk and Pasterkamp, 2019).

Mutations in the gene for FUS (fused in sarcoma) make up approximately 5% of hereditary ALS cases (Kwiatkowski et al., 2009; López-Erauskin et al., 2018; Vance et al., 2009). The FUS protein has a RNA/DNA recognition motif, a putative nuclear export signal, several disorganized domains, and a nuclear localization signal at the C-terminus (Kino et al., 2011; Lorenzo-Betancor et al., 2014; Murakami et al., 2012). FUS has been associated with a plethora of cellular functions, including translation, splicing, RNA transport, and DNA damage response (Andersson et al., 2008; Lagier-Tourenne et al., 2010; Ratti and Buratti, 2016; Therrien et al., 2016; Wang et al., 2018). Under physiological conditions, FUS is predominantly located in the nucleus, but it is able to shuttle between nucleus and cytoplasm in response to different stimuli (Gal et al., 2011; Vance et al., 2013). Pathological mutations are suggested to result in toxic gain of function, as ALS-associated mutant FUS is prone to cytoplasmic accumulation. The potential role of loss of function due to FUS sequestration in the cytoplasm is still being debated (An et al., 2019). Currently, mechanisms by which mutant FUS proteins lead to motor neuron degeneration are still largely enigmatic. A prevailing hypothesis is that mutated FUS proteins form irreversible hydrogels that impair ribonucleoprotein (RNP) granule function (Murakami et al., 2015).

In a previous study, several variants of human ALS-associated FUS were ectopically expressed in the *Caenorhabditis elegans* (MAUPAS 1900) nervous system (Murakami et al., 2012). These worms died prematurely, and had impaired motility, suggesting degeneration of the locomotor system through a gain-of-function mechanism. Strength of these phenotypes was positively correlated with the severity of human ALS caused by each variant, supporting *C. elegans* as a promising experimental paradigm to interrogate mechanisms by which mutant FUS proteins lead to structural and functional disruption of the nervous system. Here, we used behavioral, ultrastructural, and electrophysiological approaches to investigate how ALS-associated FUS impacts the *C. elegans* neuromuscular system. We show that the organization of the neuromuscular junction (NMJ) is impacted by human FUS, and ALS-associated FUS severely impairs functional communication between motor neurons and muscles. These disease mechanisms may contribute insights to other forms of ALS, as well as other neurodegenerative diseases.

## RESULTS

### Expression of ALS-associated FUS in the *C. elegans* nervous system results in reduced lifespan and impaired motility

**Fig. 1.**
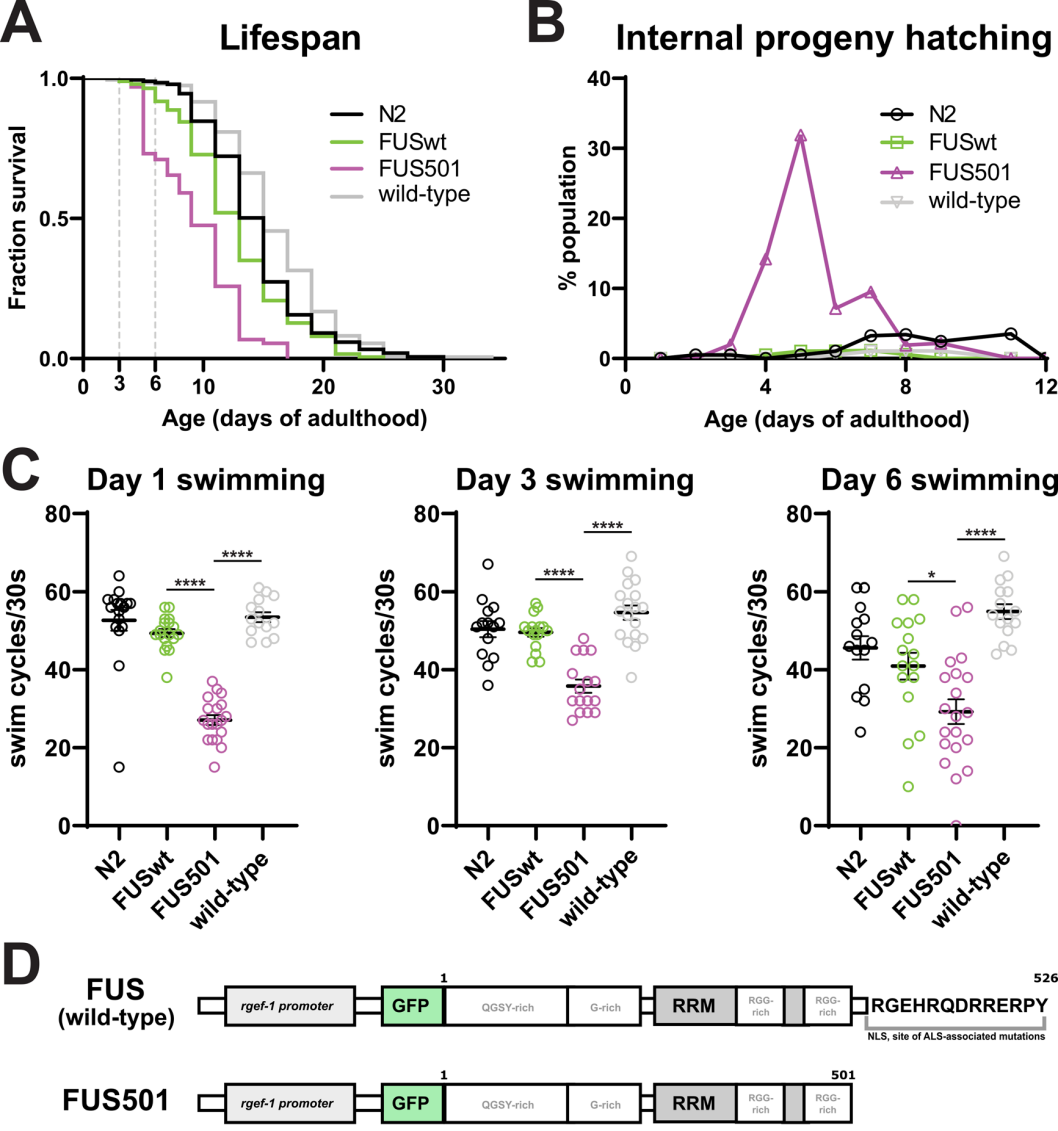
***C. elegans* expressing pan-neuronal human FUS501 exhibit lifespan and motility defects.** (A) Animals expressing human FUS501 have a shorter median and maximum lifespan compared to wild-type animals, as well as animals expressing wild-type human FUS (*n*≥102 deaths per genotype; log-rank test, *P*<0.0001). Animals expressing wild-type human FUS also displayed a mild lifespan defect (compared to N2, *n*≥160 deaths per genotype; log-rank test, *P*=0.0014). (B) Internal hatching of progeny occurred more frequently in FUS501 animals than in non-transgenic animals and animals expressing wild-type human FUS. (C) *C. elegans* expressing FUS501 have defective swimming compared to wild-type, whereas animals expressing wild-type human FUS are able to swim similarly to N2 controls (*n*≥14 per genotype; one-way ANOVA followed by Tukey's multiple comparisons, *P=0.0112; ****P<0.0001). (D) The alleles of FUS used in this study. Wild-type human FUS contains regions enriched with certain amino acids (one-letter code given), an RNA recognition motif (RRM) and a nuclear targeting signal at the C-terminus (NLS). Mutations in this region are often linked to ALS.

To determine the time window for analysis of the *C. elegans* ALS-model, we examined the hermaphrodite worms that stably and pan-neuronally express a pathological form of FUS, FUS C-terminal deletion (referred as FUS501 henceforth) (Murakami et al., 2012, 2015) for effects on lifespan and motor function. Consistent with previous results (Murakami et al., 2012, 2015), we observed a shortened lifespan of FUS501 animals compared to wild-type (N2) animals, as well as a genetically wild-type sibling strain derived from outcrossing FUS501 animals with N2 (referred to as ‘wild-type’; Fig. 1A). FUS501 animals were also more likely to die from internal hatching of progeny (a phenotype referred to as bagging), likely due to impairment of the neural circuit that modulates egg-laying (Fig. 1B). Consistent with motor dysfunction, FUS501 animals showed reduced motility in swimming assays (Fig. 1C). Animals expressing wild-type human FUS (referred to as FUSwt henceforth) displayed a milder reduction of lifespan, but did not display a motor defect at the ages tested (Fig. 1A–C).

Based on these results, we chose to proceed with comparative ultrastructural and functional analyses for FUS501 and wild-type animals on day 3 of adulthood. We reasoned that a pathological synaptic phenotype should already be pronounced prior to widespread degeneration, before causing secondary phenotypes that could make analysis more difficult. At this age, pathological features, including the FUS protein localization, aggregation, and motility defects were prominent, while animal survival was still high (Fig. 1A,C).

### Large endosomes with electron-dense inclusions are enriched at the neuromuscular junctions (NMJs) of FUS501 adults

In order to assess whether FUS501 motility defects reflect synaptic structural defects, we used electron tomography to map the organization of excitatory and inhibitory NMJs in animals expressing wild-type human FUS (FUSwt) and FUS501, as well as the non-transgenic, wild-type animals that share otherwise the same genetic background.

**Fig. 2.**
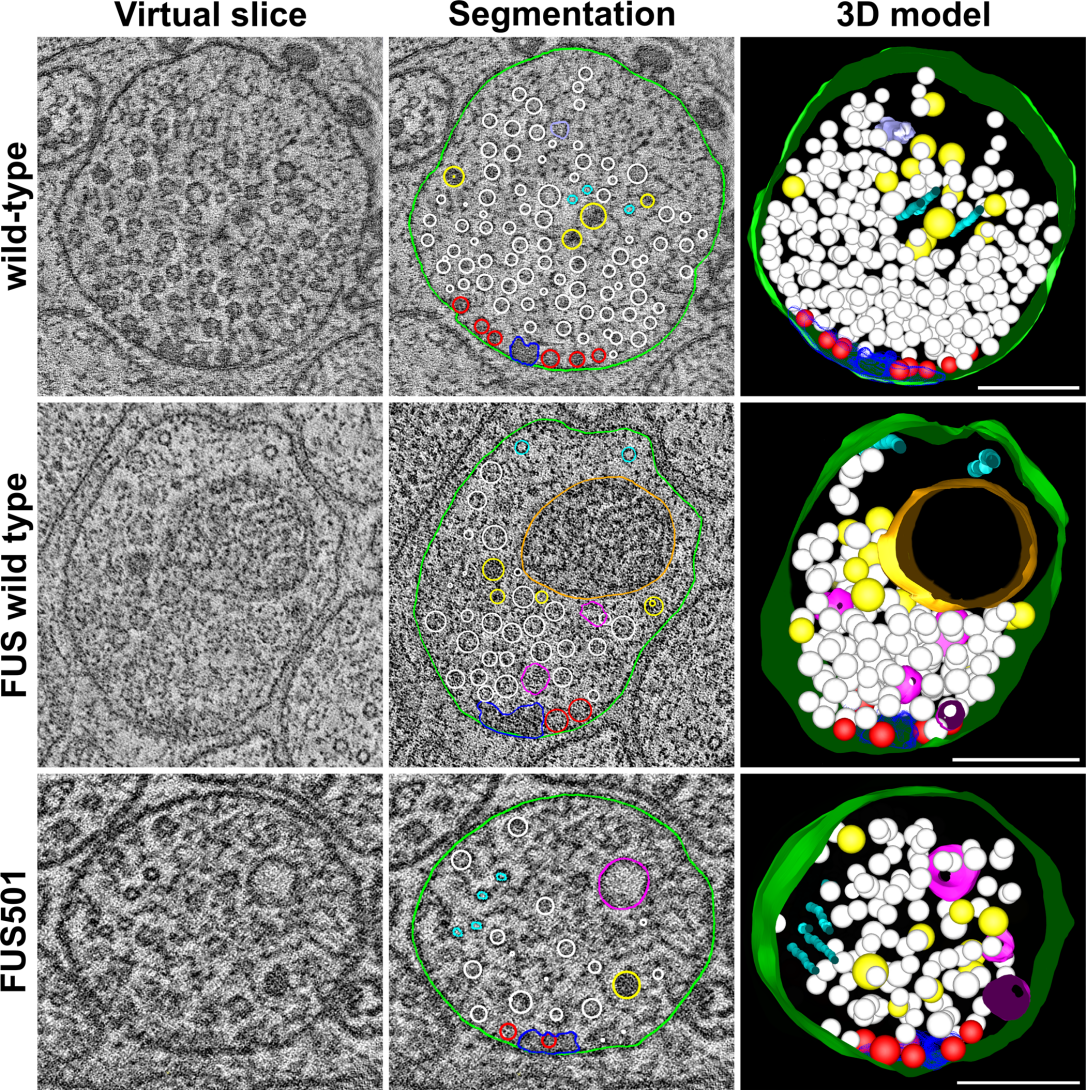
**FUS501 affects the synaptic ultrastructure of cholinergic motor neurons.** Shown are virtual ∼1 nm slices from electron tomograms of cholinergic motor neurons, segmentations of these slices, and the 3D models of the whole tomograms. Segmented structures: plasma membrane (green), mitochondria (orange), dense projections (dark blue), microtubules (cyan), endoplasmic reticulum (lavender), dense core vesicles (yellow), clear core vesicles (white), docked clear core vesicles (red), and endosome-like structures (pink). Large, endosome-like structures appear in synapses affected by mutated FUS501, but not in FUSwt and wild-type controls. FUSwt controls show smaller structures that presumably represent normal endosomes. Scale bars: 200 nm.

**Fig. 3.**
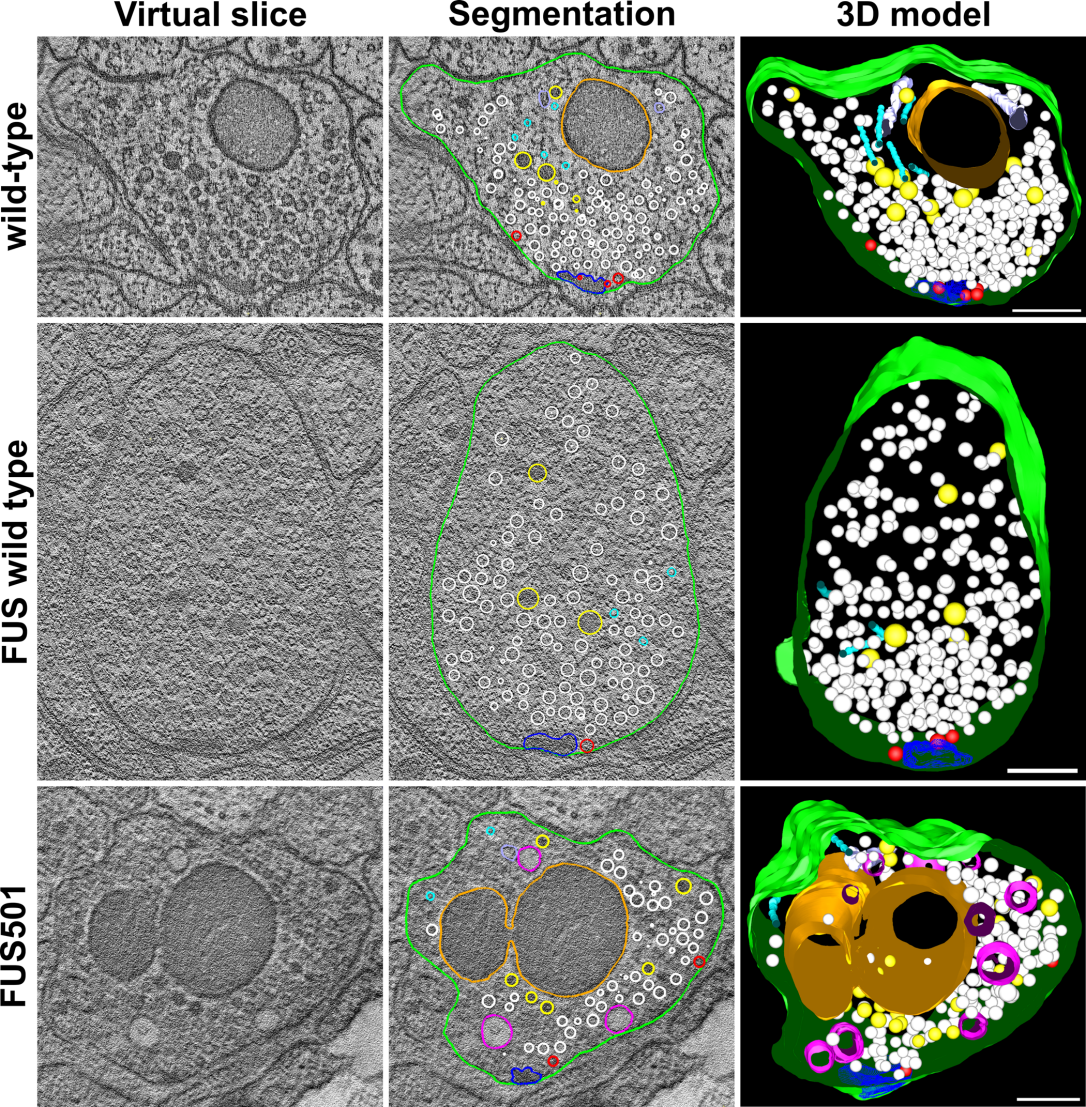
**FUS501 affects the synaptic ultrastructure of GABAergic motor neurons.** Shown are virtual ∼1 nm slices from electron tomograms of GABAergic motor neurons, segmentations of these slices, and the 3D models of the whole tomograms. Segmented structures: plasma membrane (green), mitochondria (orange), dense projections (dark blue), microtubules (cyan), endoplasmic reticulum (lavender), dense core vesicles (yellow), clear core vesicles (white), docked clear core vesicles (red), and endosome-like structures (pink). Large, endosome-like structures appear in synapses affected by mutated FUS501, but not in FUSwt and wild-type controls. Scale bars: 200 nm.

**Fig. 4.**
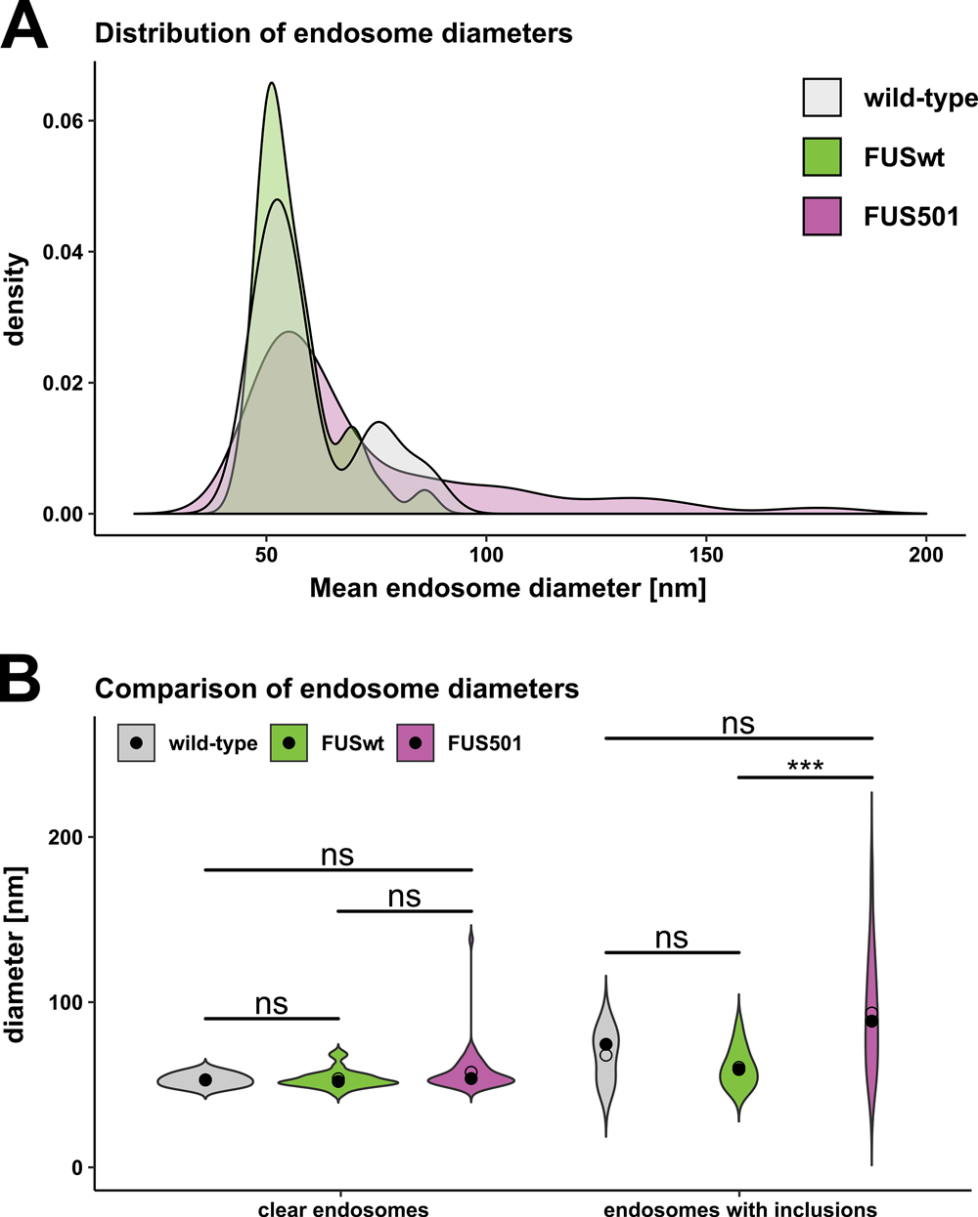
**Larger endosomes that contain electron-dense filaments are caused by FUS501.** Electron tomograms of hermaphrodite worms on day 3 of adulthood (5 for wild-type, 17 for FUSwt, and 26 for FUS501) were used to manually measure endosome diameters. For each endosome, the average diameter calculated from the longest and shortest measured diameter was used for subsequent analysis. (A) Density plot of endosome diameters. FUS501 worms show populations of especially large endosomes not present in controls (arrows). (B) Comparison of endosome diameters in relation to the presence of electron-dense filaments. Statistical analysis via Mann–Whitney–Wilcoxon test. Data are depicted as violin plots. Median (closed circles) and mean (open circles) are given on each plot. For details see Materials and Methods and Table 1.

**Fig. 5.**
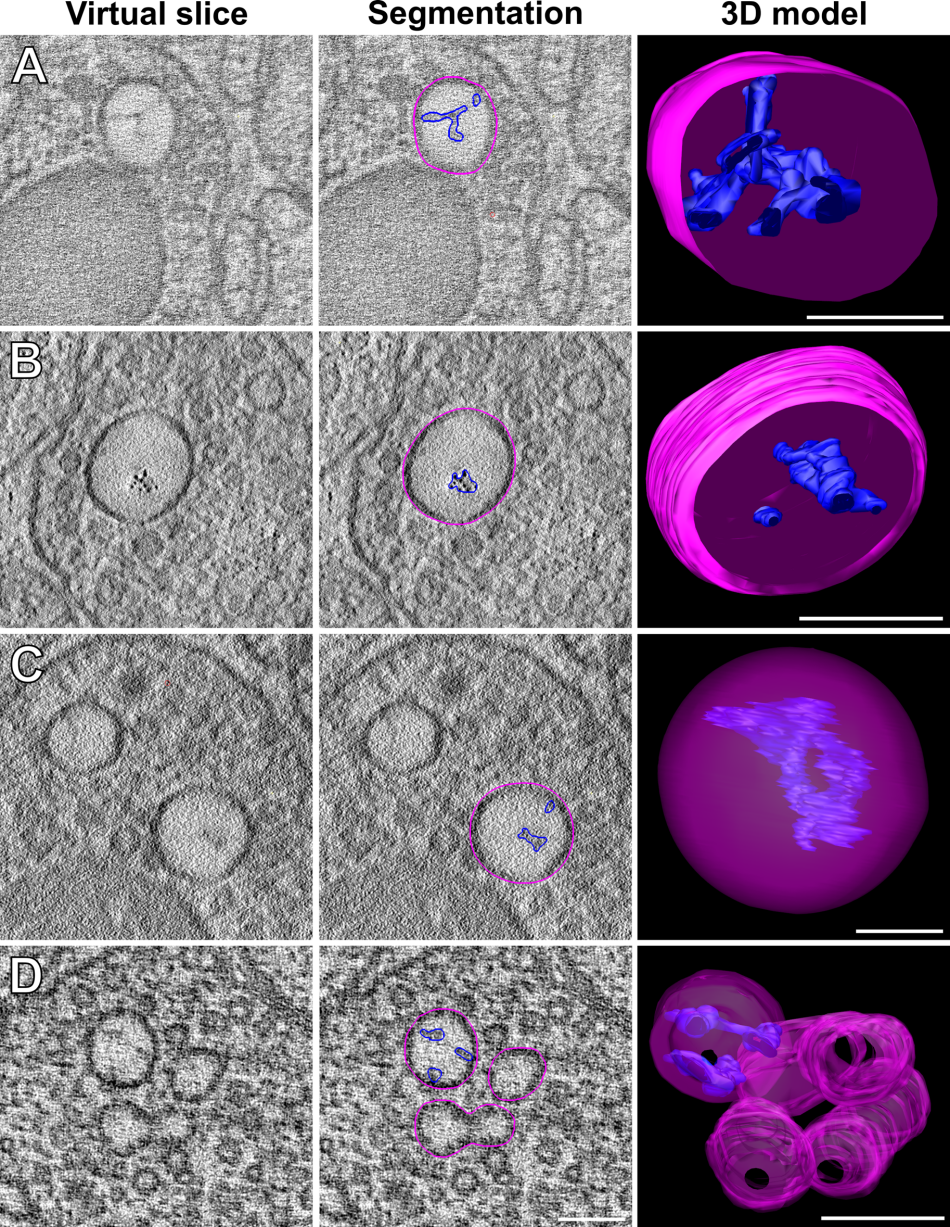
**Morphology of the large endosomes in FUS501.** Examples of large endosomes (pink) in synapses of NMJs. Only endosomes in tomograms containing a dense projection are shown here. They appear large, with diameters of >80 nm, and often contain some electron-dense material (dark blue). (A) Typical example. The electron-dense content is often branched, as shown. (B) Electron-dense content appears partially in distinct dots. (C) Instance of a complete large endosome. (D) In one instance, large endosomes formed a group and ‘network’ as shown. Scale bars: (A, B, D): 100 nm, (C): 50 nm. For details see Materials and Methods and Table 1.

Wild-type and FUSwt NMJs were composed of a presynaptic dense projection and associated pool of clear and dense core vesicles, and rarely contained large endosomes. However, large endosome-like organelles were enriched at the NMJs of animals expressing FUS501, and a population of large endosomes (>100 nm) was exclusively present in FUS501 worms (Figs 2–4). This was accompanied by a reduction in the smaller (∼50 nm) diameter endosomes in FUS501 (Fig. 4A). These endosome-like organelles were mostly electron-light, and present in both cholinergic and GABAergic NMJs (Figs 2 and 3). Most endosomes were spherical, although more complex networks also occurred (see Fig. 5; Fig. S1).

**Table 1.**
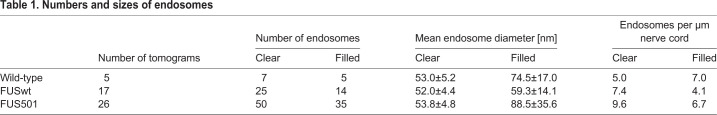
**Numbers and sizes of endosomes**

In FUS501 mutants, approximately 41% of endosome-like structures had filamentous, electron-dense inclusions (35/85, from 26 tomograms). Wild-type and FUSwt animals also had a similar percentage of filled endosomes (36% and 41% respectively; 5/12 from 5 tomograms, and 14/39 from 17 tomograms). While ‘empty’ endosomes in each genotype were of a similar diameter (*P*-values: FUS501 versus FUSwt: 0.089; FUS501 versus wild-type: 0.24; FUSwt versus wild-type: 1.0), endosomes in FUS501 worms containing electron-dense inclusions were larger than similar filled endosomes in wild-type and FUSwt animals (Fig. 4B, Table 1).

The statistical difference in endosome diameter between FUSwt and FUS501 was highly significant (*P*-value=0.00074), but only a tendency between wild-type and FUS501 (*P*-value=0.098) (Fig. 4B). There was no significant difference between wild-type and FUSwt (*P*-value=0.40). The low number of endosomes analyzed for wild-type might explain the lack of statistical significance. Nevertheless, these data suggest that FUS501 expression in the *C. elegans* nervous system results in the emergence of a population of large endosome-like structures at the NMJ, likely through enlargement of endosomes that contain electron-dense filaments.

### The size of vesicles at the NMJ is modified by human FUS

**Fig. 6.**
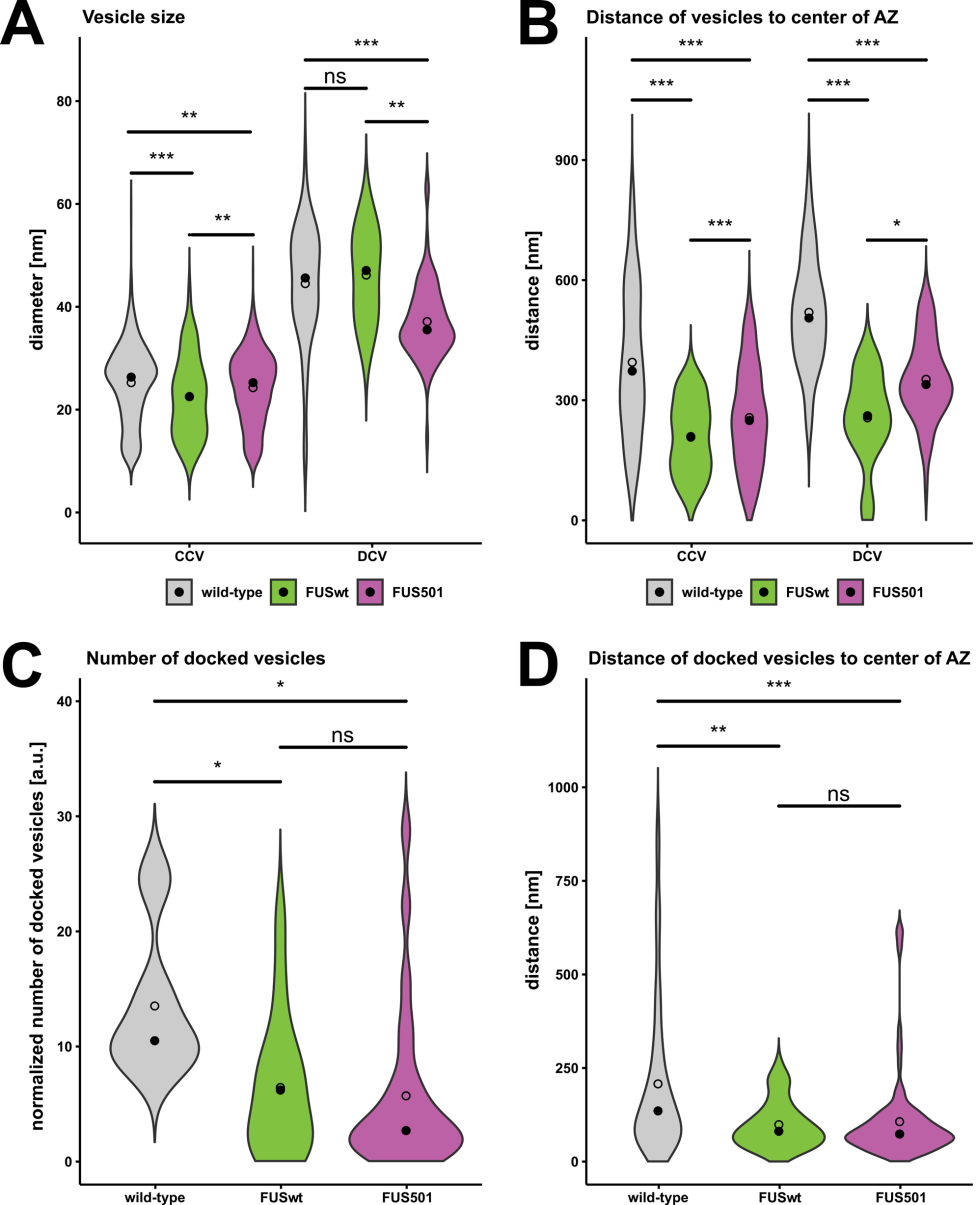
**Expression of human FUS modifies the size, position, and docking of vesicles at NMJs.** Electron tomograms of hermaphrodite worms on day 3 of adulthood (wild-type, FUSwt, or FUS501) were used for automated and manual analysis. Only cholinergic synapses were included. Statistical analysis via Mann–Whitney–Wilcoxon test. Data are depicted as violin plots. Median (closed circles) and mean (open circles) are given on each plot. (A,B) Vesicle reconstruction and classification via the 3D ART VeSElecT and automated classification Fiji macros (Kaltdorf et al., 2017, 2018). In total, 2053 vesicles at cholinergic NMJs were reconstructed for wild-type, 164 for FUSwt, and 1030 for FUS501. (A) Linear distances of the center points of all vesicles to the center of the active zone (AZ) as given by the classification macro. (B) Vesicle radii as given by the classification macro. (C,D) Analysis of vesicles docked to the plasma membrane via manual analysis. (C) Numbers of docked vesicles per tomogram normalized to approximate volume of the dense projection in a given tomogram. (D) Linear distances of docked vesicles to the center of the AZ. For details see Materials and Methods and Table 2.

Given the presence of large endosomes in FUS501 terminals, we used automated classification tools (Kaltdorf et al., 2017, 2018) to reconstruct the vesicle pools from tomograms of cholinergic NMJs. We found that clear synaptic vesicles (CCVs) had similar diameters between the three genotypes, although the small differences were statistically significant (wild-type: 26.4±7 nm; FUSwt: 22.6±8.8 nm; FUS501: 25.2±7 nm; *P*-values: FUSwt versus wild-type: 8.9e-06; FUS501 versus FUSwt: 0.0024; FUS501 versus wild-type: 0.0049; Fig. 6A; Table 2). For dense core vesicles (DCVs), the diameter was similar between wild-type and FUSwt (wild-type: 45.6±10.6 nm; FUSwt: 47±10.6 nm; Table 2), but was significantly smaller in FUS501 (35.6±5.6; *P*-values: FUS501 versus FUSwt: 0.0074; FUS501 versus wild-type: 4.0e-08; Table 2). DCVs made up 4.5% of all vesicles in wild-type, 4.9% in FUSwt, and 11.4% in FUS501 (*P*-values of two-sample *t*-tests: FUSwt versus wild-type: 0.83; FUS501 versus FUSwt: 0.012; FUS501 versus wild-type: <0.00001). Thus, in FUS501 worms, vesicle pools in NMJs contain a higher proportion of DCVs that are smaller in size (Fig. 6A).

**Table 2.**
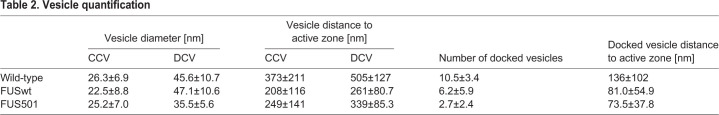
**Vesicle quantification**

### FUS disrupts the organization and docking of vesicles at the NMJ

We further compared the distance between vesicles and the center of the presynaptic dense projection. In animals expressing FUSwt and FUS501, both clear- and dense-core vesicles were closer to the presynaptic dense projection than wild-type controls (Fig. 6B; Table 2). For both classes of vesicles, the effect of FUSwt was more pronounced than FUS501 (Fig. 6B; Table 2). To assess how altered distribution of vesicles may affect their release properties, we quantified the number of docked vesicles at each synapse. Both FUSwt and FUS501 had significantly fewer docked vesicles than wild-type controls (*P*-values: FUSwt versus wild-type: 0.025; FUS501 versus FUSwt: 0.44; FUS501 versus wild-type: 0.011; Fig. 6C; Table 2). Docked vesicles in FUSwt and FUS501 were closer to the center of the presynaptic dense projection than wild-type controls, similar to the global vesicle distribution described earlier (Fig. 6D; Table 2). Thus, in FUSwt and FUS501, fewer vesicles are docked, but those that are docked located closer to the presynaptic dense projection.

### FUS501 animals exhibit reduced endogenous postsynaptic currents

**Fig. 7.**
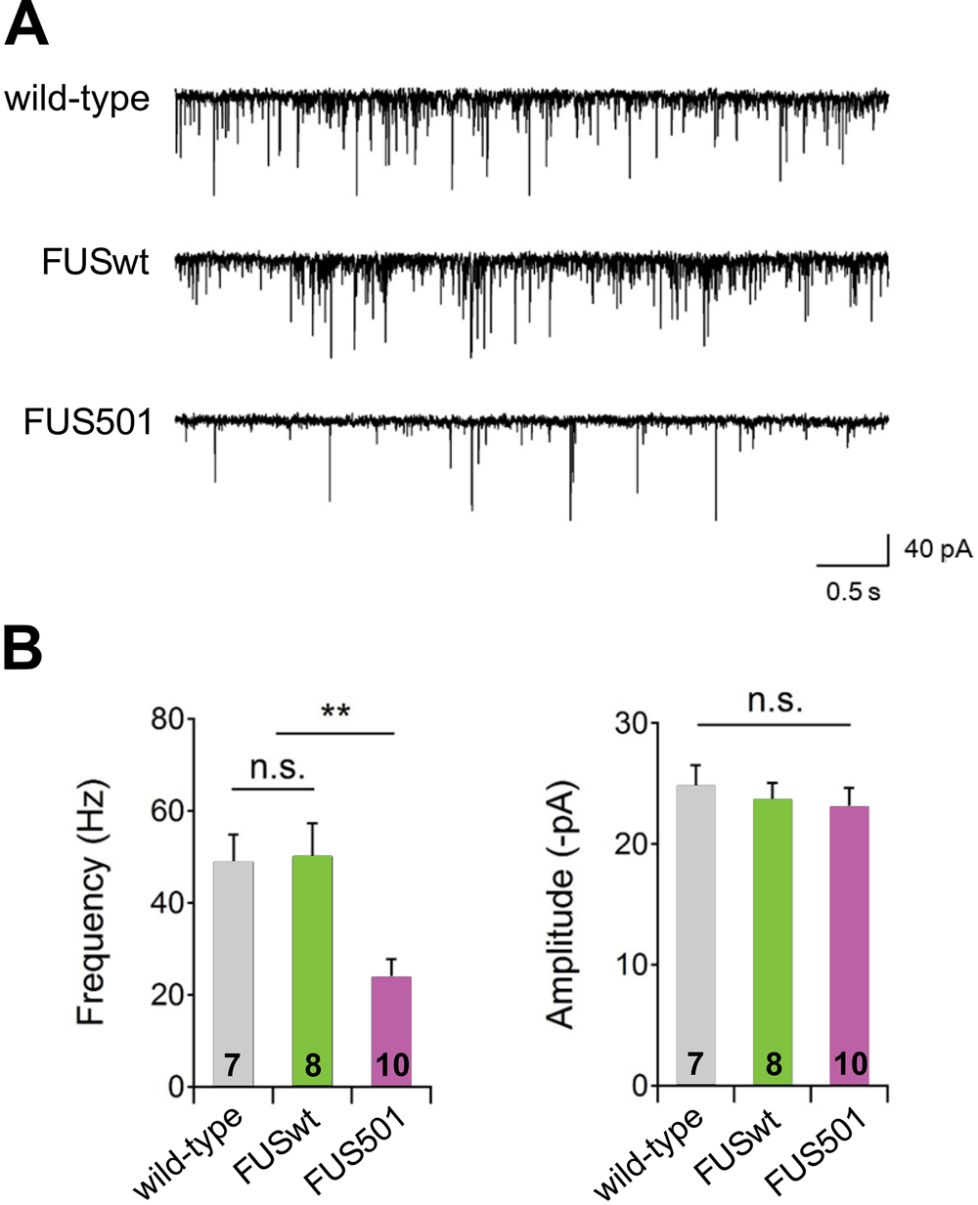
**The frequency of endogenous postsynaptic currents is decreased in FUS501 transgenic animals.** (A) Representative traces of endogenous postsynaptic currents in wild-type, FUSwt, and FUS501 animals. Muscles were held at -60 mV. (B) The frequency of endogenous postsynaptic currents was significantly decreased in FUS501 compared to wild-type or FUSwt animals. (C) The amplitude of endogenous postsynaptic currents showed no significant difference between strains.

Given the defects in the structural organization of NMJ in animals expressing human FUS, we recorded the functional output of the NMJ by patch clamping muscle cells in animals on day 3 of adulthood. The endogenous postsynaptic currents (enPSCs) reflect synaptic vesicle fusion events that are due to either spontaneous release or release evoked by endogenous activity in the motor neurons or upstream circuits, and subsequent detection of neurotransmitter (acetylcholine or GABA) by receptors on the body wall muscles (Richmond and Jorgensen, 1999). The frequency of enPSCs was not different between the wild-type controls and FUSwt, whereas FUS501 animals exhibited a >50% reduction in enPSC frequency (Fig. 7A,B). There was no statistical difference in the enPSC amplitude among the three genotypes (Fig. 7A,C). The presence of a frequency defect in the absence of amplitude defect suggests a reduction in presynaptic vesicle release from motor neurons onto muscle cells. This could be due to defective vesicle release at individual NMJs, a reduction in the number of NMJs, or a combination of both. Because there was no change in enPSC amplitude, vesicle loading, and postsynaptic reception are likely unaffected at this timepoint.

### FUS501 proteins aggregate in the nuclei and cytoplasm of motor neurons

Given that FUS501 NMJs harbor a pool of large, filament-filled endosome-like structures, we tested if the FUS501 protein was physically present at NMJs, thus could be directly and locally responsible for defects in neurotransmission.

**Fig. 8.**
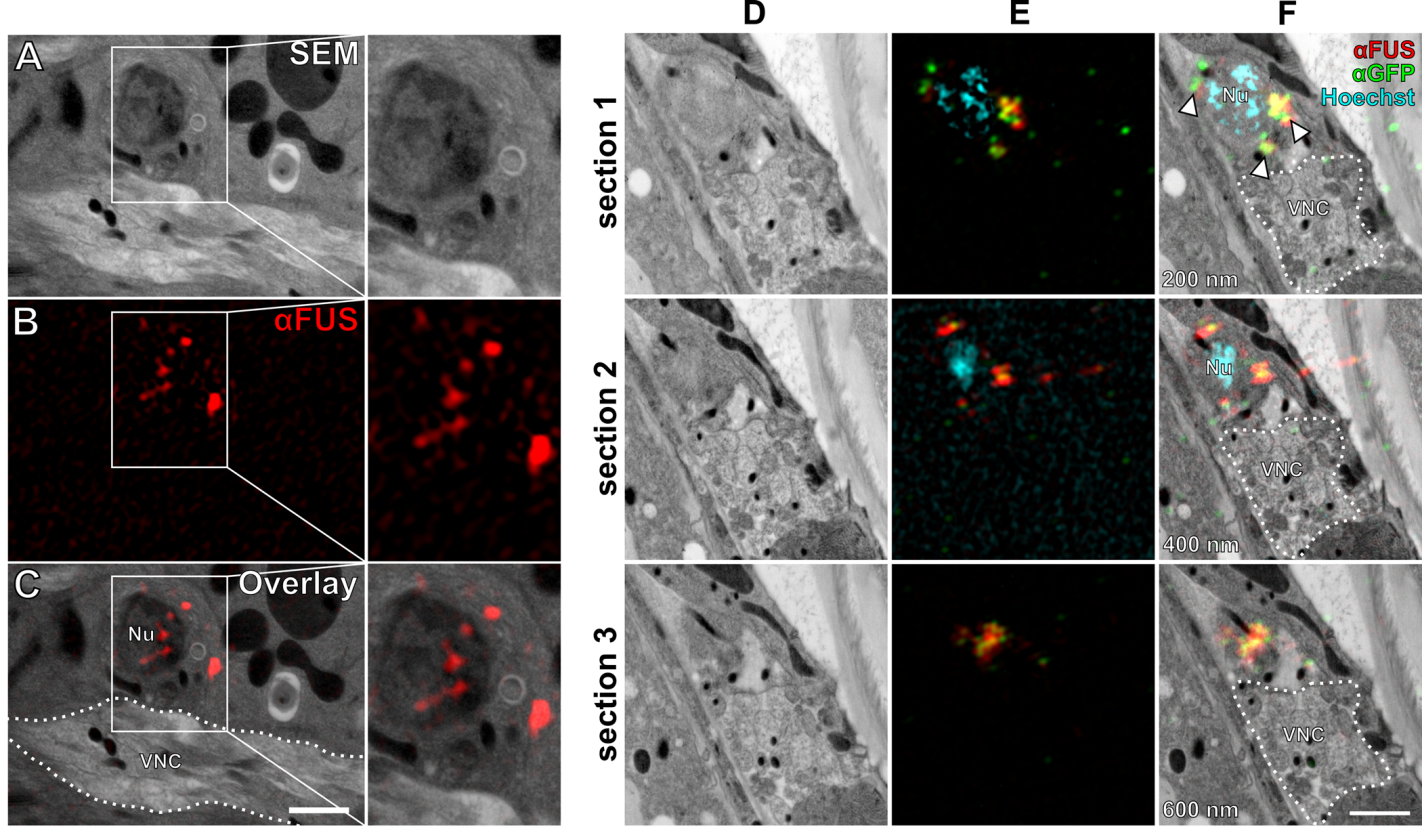
**FUS501 is present in nucleus and cytoplasm of motor neurons but not in the nerve cords.** (A) Scanning electron micrograph of the ventral nerve cord (VNC) of a FUS501 worm. (B) Immunofluorescence staining against mutated FUS acquired by SIM of the same region as in A. (C) Overlay of A and B localizes FUS501 signals to the nucleus (Nu) and cytoplasm of a motor neuron but does not show any signals on the synapses of the VNC. (D) Scanning electron micrographs of the VNC region of an adult hermaphrodite worm expressing mutated FUS. Consecutive 200 nm sections are shown. (E) Aligned SIM channels shown for reference. (F) Overlay of D and E. Hoechst was used for correlation. FUS501 was stained via direct antibody (red) and via its GFP-tag (green). Both stainings overlap significantly (arrowheads). Scale bar: 1 µm.

Light microscopy studies have suggested that FUS501 forms aggregates in motor neuron cell bodies and neurites that expand the ventral nerve cords (Murakami et al., 2012, 2015; see also Figs. S2 and S3). We used super-resolution array tomography (srAT; Markert et al., 2017) to localize FUS proteins in their ultrastructural context. FUS501 was detected as aggregated clusters in the nucleus and the cytoplasm of motor neuron soma, whereas FUSwt signals were diffuse and limited to the nucleus (data not shown). FUS501 was not in the nerve cords (Fig. 8). The lack of detection of FUS in motor neuron processes may indicate its absence, alternatively, there was insufficient preservation of small amounts of epitope.

## DISCUSSION

### FUS501-induced formation of large endosomal-like structures at NMJs

FUS501 worms had a population of unusually large vesicles at NMJs. Their cellular nature remains to be better defined, but their morphology and location are consistent with endosomes described at *C. elegans* NMJs: They are roughly spherical and located within the synaptic vesicle pool (Watanabe et al., 2013a), and they do not resemble other intracellular structures, such as autophagosomes (Meléndez et al., 2003) and the endoplasmic reticulum (ER). Autophagosomes feature double membranes, while our electron tomograms clearly show that these large vesicles have single membranes (Fig. 4). ER in our tomograms appeared as irregularly shaped tubes located at the synaptic periphery, whereas these vesicles appeared throughout synapses including regions close to the active zone. Thus, the population of large vesicles most likely represents endosomes.

Putative endosomes in each genotype, FUS501, as well as FUSwt and wild-type control animals, were either ‘empty’ or contained electron-dense filamentous inclusions. The larger population of putative endosomes in FUS501 animals selectively contained inclusions. Interestingly, in Alzheimer's disease and Down syndrome, endosomes have also been reported to be enlarged due to acceleration of endocytosis (Cataldo et al., 2008; Colacurcio et al., 2018). However, our data suggest a decrease in endocytosis, which is discussed further in the next section.

Electron-dense filamentous aggregates observed in these endosomes remain enigmatic. It is unknown whether these filaments are native structures, or if filaments in FUSwt or FUS501 contain FUS. It is also possible that such inclusions are a general aging phenotype, as they were also observed in our age matched wild-type controls. FUS501 aggregation may also promote aggregation of other proteins. It is well established that the *C. elegans* intracellular environment becomes more prone to aggregation across ageing, and this is exacerbated by the overexpression of aggregation-prone proteins (David et al., 2010; Huang et al., 2019; Walther et al., 2015).

Thus, FUS501 appears to selectively increase the diameter of endosome-like structures that contain inclusions. These endosome-like structures may be involved in bulk endocytosis, consistent with a possible defect in synaptic vesicle cycling in FUS501 nerve terminals.

### FUS501 interferes with neurotransmission

For both ultrastructural and functional analyses, we used two lines as controls in all experiments, wild-type animals derived from outcrossing the FUS501 strain with N2, and FUSwt. Overexpression of wild-type human FUS led to mild effects on some ultrastructural features of the nervous system, as well as in some aging phenotypes. But the degree of abnormality was substantially less that of FUS501, and was not consistent across all physiological phenotypes, for example, the frequency of endogenous postsynaptic currents. Such an effect, the mild phenotype upon overexpression of non-pathological form of a protein, has been observed for other neurodegenerative, ALS-included, associated genes (Cuvelier et al., 2018; Mirra et al., 2017; Mitchell et al., 2013; Moloney et al., 2018).

Expression of human FUS, whether FUSwt or FUS501, resulted in changes to vesicle size and distribution at the NMJ. It also resulted in fewer docked vesicles. Although this was observed at both FUSwt and FUS501 terminals, reduced enPSCs were only observed in FUS501. If changes in vesicle docking contribute to defects in neurotransmission at FUS501 NMJs, additional factors must be at play. These may be within the synaptic terminal (e.g. changes in coupling of synaptic vesicles to voltage-gated calcium channels) (Chang and Martin, 2016), within the motor neuron (e.g. reduced excitability) (Guo et al., 2017; Liu et al., 2016; Naujock et al., 2016), or in upstream circuits that modulate endogenous motor neuron activity. Alternatively, a reduction of NMJ number might account for this finding.

*C. elegans* possesses a FUS ortholog, FUST-1, with about 50% identity on the protein level. It was reported previously that deletions in *fust-1* caused neuronal degradation and paralysis, whereas overexpression did not have any obvious effects (Therrien et al., 2016). It is unknown if mutations in FUST-1 can cause similar phenotypes as FUS501. If not, it would not be surprising for two reasons. First, FUS501 (and other FUS pathological mutations) causes a gain-of-function phenotype (Murakami et al., 2012). Second, there are an unusually large number of RRM family proteins in *C. elegans* and many of them function redundantly (Thompson et al., 2019).

In ALS, excitotoxicity is a topic of concern with regards to neuron degeneration (Fogarty, 2019). It is thought that neurons are driven to decay by hyper-excitability for several forms of ALS (Fogarty, 2018). However, for FUS-mediated mouse model, motor neuron degeneration was reported to be preceded by hypo-excitability (Martínez-Silva et al., 2018), echoing the high heterogeneity of ALS (Hardiman et al., 2017). Our results are more consistent with the hypo-excitability model and the conclusion from the mouse FUS models (Kong et al., 2009; Martínez-Silva et al., 2018; Ruiz et al., 2010).

### srAT reveals the ultrastructural context of FUS localization

Consistent with the implication of fluorescent microscopy studies (Murakami et al., 2012, 2015) we observed aggregated FUS in motor neuron nucleus and cytoplasm by srAT. But we did not detect FUS accumulations in axons, most likely due to the low abundance of preserved epitopes, or insufficient sampling of our serial sections. A recent study in rodents reported FUS accumulation in synapses (Deshpande et al., 2019). Interestingly, in early development, FUS was predominantly found in postsynapses, but in mature neurons it was found in axon terminals. In *C. elegans*, FUS might only localize to synapses in certain developmental stages or not at all.

A previous study (Murakami et al., 2015) showed strong light-microscopy evidence that irreversible FUS hydrogels are associated with RNP granules, which have been described by electron microscopy using samples preserved by a different protocol (Biggiogera et al., 1997; Jokhi et al., 2013; Souquere et al., 2009). We could not identify RNA granules in our EM sections; visualizing ultrastructural features of FUS aggregates or the RNP granules they attach to may require different preservation protocols.

### A reduction of protein translation might account for endosome and vesicle docking defects

FUS has many functions related to DNA and RNA processing and maintenance (Ratti and Buratti, 2016), and knock-out leads to perinatal death in mice (Hicks et al., 2000). Irreversible hydrogels formed by mutated FUS impair RNP granule function. This reduces the rate of new protein synthesis (Murakami et al., 2015). This has a systemic effect on neurons, however, the effect on axons and synapses is likely particularly detrimental, since such intracellular compartments have been shown to be heavily dependent on local translation regulation by RNP granules in mouse (Akbalik and Schuman, 2014; Holt and Schuman, 2013; Jung et al., 2014) and in *C. elegans* (Yan et al., 2009). Furthermore, the replacement of murine FUS with a mutated human form activates an integrated stress response, and inhibits local intra-axonal protein synthesis in hippocampal neurons and sciatic nerves, resulting in synaptic dysfunction (López-Erauskin et al., 2018). A recent study found that transcription of an acetylcholine receptor is compromised in FUS-mediated ALS, thus supporting the idea that FUS affects transcription and transcript processing (Picchiarelli et al., 2019).

It is plausible that the ultrastructural defects that we found are caused by reduced protein synthesis. Under physiological conditions, vesicles are recycled via the ultrafast endocytosis pathway (Watanabe et al., 2013a,b). After endocytosis, vesicles are regenerated in a clathrin-dependent manner (Watanabe et al., 2014). Shortage of clathrin or other components of this pathway could cause an accumulation of endocytosed membrane represented by large endosomes. Our observation that median distance of vesicles to the active zone is reduced in FUS worms and a reduction of vesicle pool size is consistent with this hypothesis.

In conclusion, we have shown synaptic architecture and functional changes in worms expressing FUS501. Our results implicate a direct or indirect role of human FUS in the organization of synaptic vesicles and synaptic transmission from motor neurons to muscles. These phenotypic analyses of the *C. elegans* ALS model can aid the elucidation of cellular mechanisms that contribute to the ALS disease.

## MATERIALS AND METHODS

### Worm strains

All *C. elegans* worms were maintained according to standard methods (Brenner, 1974). The transgenic animals used in this study were ZM9566 {*hpIs239*[Prgef-1::GFP::fus(del501)]; plasmid pJH2392}, which ectopically and panneuronally expresses FUS501, and ZM5838 {hpIs223[Prgef-1::GFP::fus(wt)]; plasmid pJH2382}, which ectopically and panneuronally expresses wild-type FUS, both under the control of the *Prgef-1* promoter. The wild-type control was ZM9569, derived by selecting wild-type siblings from the final outcross of the parent FUS501 strain with N2 Bristol. Since generating transgenic animals inevitably creates background mutations, and aging might be sensitive to accumulative effect of silent mutations, ZM9569 represents a more appropriate control to compare with the recovered ZM9566 FUS501 strain from the same set of crosses. ZM9566 and ZM5838 both feature multiple copy insertions. ZM9566 and ZM5838 were both outcrossed six times to remove any potential background mutations (Murakami et al., 2012). In addition, we included the N2 Bristol strain as an additional control in the lifespan, bagging, and swimming assays.

### Lifespan

*C. elegans* were synchronized at the L4 stage and manually picked to fresh plates every 1–2 days to prevent contamination by progeny. Animals that were immotile and did not respond to gentle prodding with a platinum wire were scored as dead. Animals that bagged (died due to internal hatching of progeny) or crawled up the walls of the plate and desiccated were censored from the lifespan analysis.

### Swimming assay

In swimming experiments, animals cultured one day after the L4 larval stage were scored as day 1 adults. Ten animals at a time were transferred into a PDMS chamber on a glass slide containing 0.5 ml of M9 buffer. Swimming was recorded for 30 s using a Basler acA2500-60 μm camera mounted on a dissection microscope. A single swim cycle was defined as a complete sinusoidal movement through the head and tail.

### High-pressure freezing

The samples are subjected to >2100 bars of pressure and cooling rates of >20,000 K s^−1^. All samples used in this study were cryo-immobilized using an EM HPM100 (Leica Microsystems) high-pressure freezing machine. The procedure was to use freezing platelets (Leica Microsystems) with 100 µm recesses. They were slightly overfilled with OP50 paste (see below) and then worms were transferred into the platelet. A second platelet with a flat surface was placed on top as a lid. The samples were processed and then stored in liquid nitrogen until freeze-substitution.

### *E. coli* OP50 bacteria paste

A 100 ml volume of *E. coli* OP50 overnight culture was pelleted at 1500× ***g***, washed with 400 µl 20% bovine serum albumin (BSA) in M9 (Stiernagle, 2006) (3.0 ***g*** KH_2_PO_4_, 6.0 ***g*** Na_2_HPO_4_, 0.5 ***g*** NaCl, 1 ml 1 M MgSO_4_, H_2_O to 1 l; sterilize by autoclaving), spun down again, and carefully re-suspended in 20 µl 20% BSA in M9.

### Freeze-substitution and resin embedding for structural analyses

The protocol is based on (Weimer, 2006). A description of the individual steps of the freeze-substitution and resin embedding can be found in (Stigloher et al., 2011). We used Epon instead of Araldite in this study.

### Freeze-substitution and resin embedding for srAT analyses

The protocol is also based on (Weimer, 2006). A detailed description of the individual steps of the freeze-substitution and resin embedding can be found in (Markert et al., 2017).

### Ultramicrotomy for srAT

A detailed description of the individual steps can be found in (Markert et al., 2017). Briefly, 100 nm sections were produced using a special diamond knife with a boat large enough to accommodate glass slides (histo Jumbo diamond knife, DiATOME). Slides were submerged in the boat before sectioning. Then the desired number of sections was cut without interruption. If necessary, a long ribbon was carefully divided into smaller ribbons using two mounted eyelashes.

### Ultramicrotomy for electron tomography

For imaging with a 200 kV TEM, we used sections up to 250 nm with good results. Sections between 150 and 200 nm in thickness were favored.

### Immunostaining

Ultrathin sections of LR White-embedded tissue were immunostained for srAT. Ultramicrotomy exposed epitopes at the section surface. Thus, sections could be stained, even though antibodies do generally not penetrate the resin.

A detailed protocol can be found in (Markert et al., 2017). In brief, sections were placed in a humid chamber, and blocking buffer was applied to the sections prior to staining with primary and secondary antibodies. They were washed with buffer and then stained with a DNA stain, where applicable. Lastly, sections were mounted with Mowiol and stored at 4°C for up to a week before fluorescence imaging.

We used a polyclonal antibody against GFP (Abcam, catalog number: ab13970) at a dilution of 1:500 and a polyclonal antibody against FUS (Bethyl, A300-293A) at a dilution of 1:1000.

### Imaging for srAT

The workflow of srAT imaging has been published in detail (Markert et al., 2016, 2017).

### Preparation of sections and imaging for electron tomography

Imaging was performed with the SerialEM (Mastronarde, 2005) and IMOD (Kremer et al., 1996) software packages. A 200 kV JEM-2100 (JEOL) electron microscope equipped with a TemCam F416 4k×4k camera (Tietz Video and Imaging Processing Systems) was used for all TEM imaging and electron tomography.

### Contrasting

Contrasting was achieved by floating the grids sections down on drops of 2.5% uranyl acetate in ethanol for 15 min and 50% Reynolds’ lead citrate (Reynolds, 1963) in ddH_2_O for 10 min. During incubation, samples were covered to minimize evaporation. During incubation in lead citrate, sodium hydroxide pellets were placed around the samples to decrease local carbon dioxide concentration. Carbon dioxide forms precipitates with lead citrate. In between contrasting steps, the grids were washed first in ethanol, then in 50% ethanol in ddH_2_O, and finally in ddH_2_O. After contrasting, they were thoroughly washed in ddH_2_O and blotted dry with filter paper.

### Carbon coating and placement of gold fiducials

Grids used for electron tomography were coated with a thin layer of carbon to prevent charging during imaging at high tilt angles. The carbon layer had an approximate thickness of 3 nm.

Gold fiducials were used to facilitate tomogram reconstruction. To achieve fiducial placement, a non-specific antibody conjugated with 10 nm gold particles was used. The antibody was diluted 1:10 with ddH_2_O and 50 µl of this dilution were pipetted on a piece of clean parafilm. The carbon-coated grids were then floated on the drop for 10 min on each side, with a single wash in ddH_2_O in between and at the end. A single wash meant that the grid was submerged in water for 1 second and then immediately dried with a filter paper. The gold fiducial placement was always performed right after carbon coating or at most a few hours later. For unknown reasons, longer delays caused very pronounced electron-dense precipitation on the sections, making them unsuitable for imaging in extreme cases.

### Acquisition of tilt series

Tilt series for this thesis were acquired either from 60° to -60° or from 70° to -70°. Double tilts were performed where appropriate and possible, i.e., tilt series from a region of interest were acquired in two orthogonal tilt axes. This was achieved by manually rotating the grid by about 90° in the sample holder. Double tilts improved tomogram quality significantly. They were not performed when the tomogram of a single axis was sufficient to answer the specific questions.

### Tomogram reconstruction

All tomograms were reconstructed with the eTomo software from the IMOD package (Kremer et al., 1996). Gold fiducials were always included to improve the alignment of the tilt series. For the step of tomogram positioning, the option ‘find boundary model automatically’ was used. Manual adjustments of the boundary model were almost never necessary. Tomograms were always created using the ‘Back Projection’ algorithm.

### Segmentation and 3D reconstruction

Segmentation and 3D reconstruction were performed with the 3Dmod software from the IMOD package (Kremer et al., 1996). Investigators were blinded regarding the genotype. All structures except for the vesicles were segmented as closed objects using the ‘sculpt’ tool. Clear core and dense core vesicles were annotated as perfect spheres by creating a point in the center of a vesicle using the ‘normal’ drawing tool. This point was then resized with the mouse wheel to match the outer dimensions of the given vesicle. Global quality of points was set to 4 to achieve smooth spheres and the ‘drawing style’ of points was set to ‘fill’ to obtain closed surfaces. All other objects except the dense projections were meshed to obtain closed surfaces here as well. Dense projections were left with the default drawing style ‘lines’. The ‘interpolator’ tool was used whenever appropriate. For large structures like the plasma membranes, gaps of 20 virtual sections or more were linearly interpolated. For mitochondria and microtubules typically gaps of ten sections were interpolated. Larger spherical structures like endosomes were interpolated with the ‘spherical’ option. Dense projections were not interpolated.

### Quantitative analyses

Automatic vesicle reconstruction from electron tomograms was performed via macros for the open source image processing software Fiji (Schindelin et al., 2012) as described in (Kaltdorf et al., 2017). They were then automatically classified into clear core and dense core vesicles according to (Kaltdorf et al., 2018). Manual adjustments of the outcomes were not performed. However, if overall classification results for a given tomogram were not satisfactory, this tomogram was excluded from analysis.

The active zone was determined manually for the classification macro. A point on the plasma membrane that is closest to the center of gravity of the dense projection seen in a given tomogram was set as the center of the active zone. The center of gravity of the dense projection was chosen by visual judgment of the user during the macro workflow.

Manual vesicle reconstruction from electron tomograms was performed via 3Dmod from the software package IMOD (Kremer et al., 1996). The center points of vesicles were set by the user's judgment and set as centers of spheres with the approximate outer diameter of the vesicles.

The dense projections were segmented manually, and their center of gravity was determined with the ‘imodinfo’ function of IMOD. The center of the active zone was defined as the intersection of the inner plasma membrane and an orthogonal line through the center of gravity of the dense projection. Linear distances of vesicles to the active zone were measured with the ‘measure’ tool in 3Dmod from the centers of the vesicles to the center of the active zone.

Average endosome diameters were calculated from the longest and shortest diameter of a given endosome measured manually on the virtual tomogram slice where the endosome appeared largest.

### Statistical analyses

For vesicles and endosomes statistical analyses and their representations were performed with R (R Core Team, 2017). Kruskal–Wallis tests (one-way ANOVA on ranks; normality of the data is not assumed) determined if samples originated from the same distributions. The Mann–Whitney­–Wilcoxon test was then used to determine statistical significance of differences between pairs in the groups. For the survival assay, log-rank tests were used. For the swimming assay we performed unpaired two-tailed Student's *t*-tests. The following significance levels were applied: *P<0.05, **P<0.01, ***P<0.001.

Variability of quantitative data samples was measured via median absolute deviation (MAD). The MAD is more robust against outliers and suitable for non-parametric data, i.e., data that does not show normal distribution (Pham-Gia and Hung, 2001). It is defined as the median of the absolute deviations of the data's median:
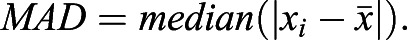
for a univariate dataset *x*_1_, *x*_2_, …, *x_n_* where *x̄* is the median of the data: 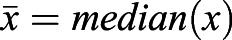.

### Electrophysiology

The dissection of the *C. elegans* was described previously (Richmond and Jorgensen, 1999). Briefly, hermaphrodites on day 3 of adulthood were glued to a PDMS-coated cover glass covered with bath solution. The integrity of the ventral body muscle and the ventral nerve cord were visually examined via DIC microscopy, and muscle cells were patched using fire-polished 4–6 MΩ resistant borosilicate pipettes (World Precision Instruments, USA). Membrane currents were recorded in the whole-cell configuration by a Digidata 1550B and a MultiClamp 700B amplifier, using the Clampex 10 software and processed with Clampfit 10 (Axon Instruments, Molecular Devices, USA). Data were digitized at 10–20 kHz and filtered at 2.6 kHz.

The recording solutions were as described in our previous studies (Gao and Zhen, 2011). Specifically, the pipette solution contains (in mM): K-gluconate 115; KCl 25; CaCl_2_ 0.1; MgCl_2_ 5; BAPTA 1; HEPES 10; Na_2_ATP 5; Na_2_GTP 0.5; cAMP 0.5; cGMP 0.5, pH7.2 with KOH, ∼320 mOsm. The bath solution consists of (in mM): NaCl 150; KCl 5; CaCl_2_ 5; MgCl_2_ 1; glucose 10; sucrose 5; HEPES 15, pH7.3 with NaOH, ∼330 mOsm. Leak currents were not subtracted. All chemicals were from Sigma-Aldrich. Experiments were performed at room temperatures (20–22°C)

## Supplementary Material

Supplementary information
