# Supplementary material for: Overexpression of an ALS-associated FUS mutation in C. elegans disrupts NMJ morphology and leads to defective neuromuscular transmission
Source: Biol Open. 2020 Dec 7;9(12):bio055129. doi: 10.1242/bio.055129 (PMC7746668; doi:10.1242/bio.055129)
Supplement: Supplementary information [file biolopen-9-055129-s1.pdf]

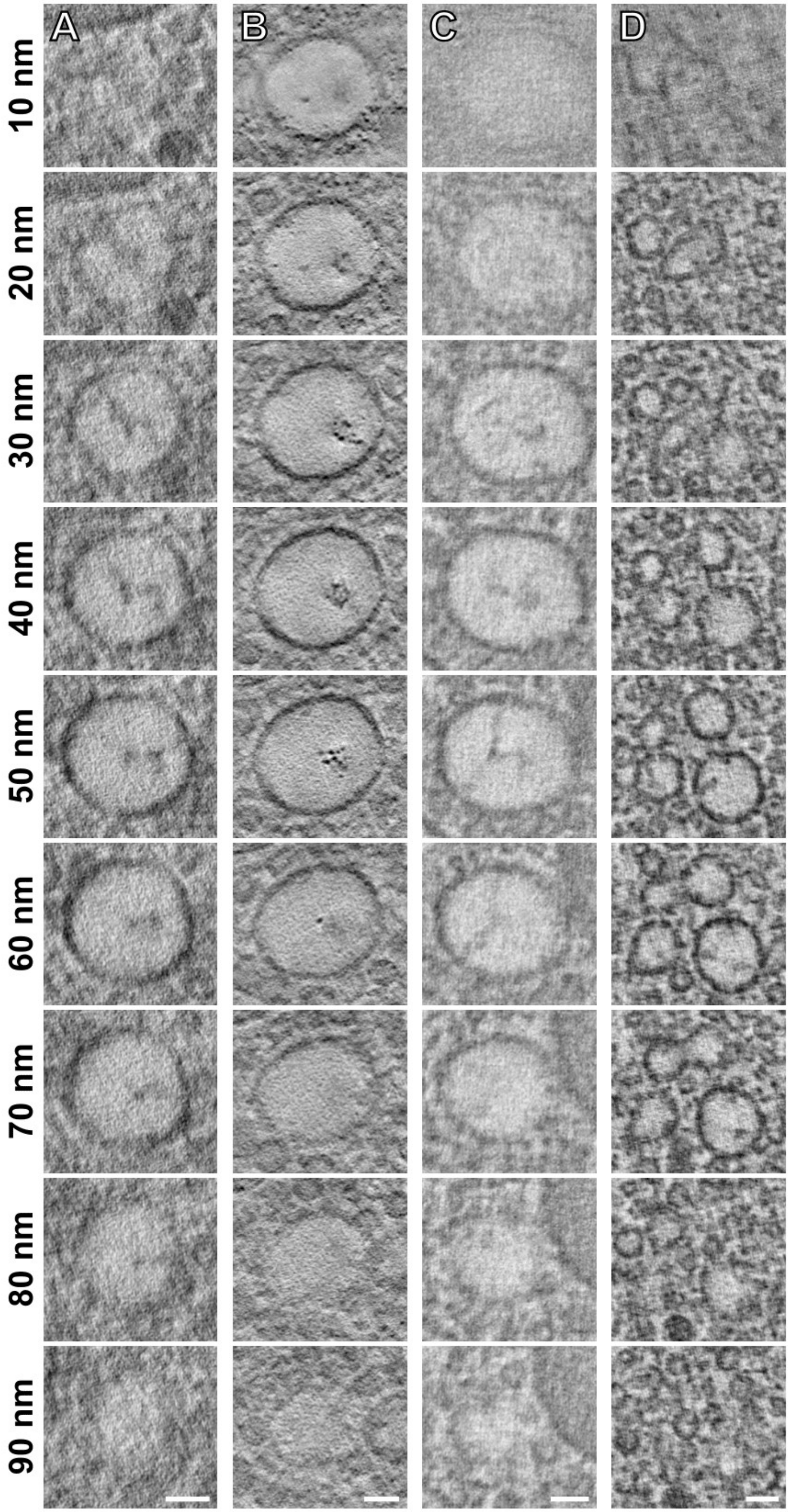
A
B
C
D
10 nm
20 nm
30 nm
40 nm
50 nm
60 nm
70 nm
80 nm
90 nm

Figure S1: Virtual consecutive 10-nm sections of the endosomes shown in Figure 5. Virtual sections were produced from electron tomograms of FUS501 synapses. Scale bars: 1 μm.

Biology Open • Supplementary information

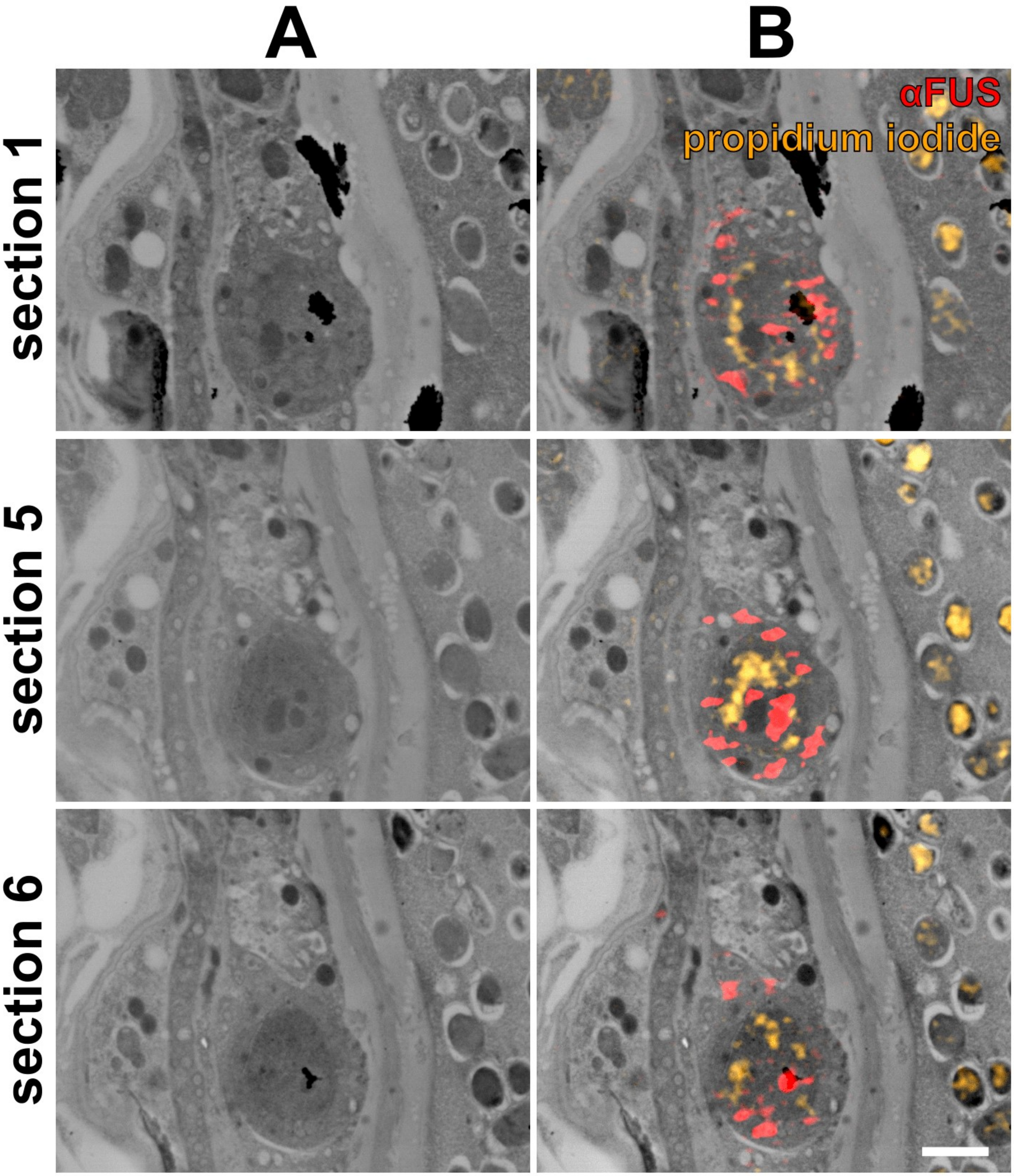

Figure S2: A selection of sections through a VNC consistently shows localization of mutated FUS in both cytoplasm and nucleus. (A) Scanning electron micrographs of the VNC region of an adult hermaphrodite worm expressing mutated FUS. (B) The images of (A) overlaid with their corresponding fluorescent SIM images. Propidium iodide was used for correlation. FUS501 signals are consistently localized to both the nucleus and the cytoplasm of a motor neuron. The patchy staining suggests the presence of FUS accumulations. Scale bar: 1 μm.

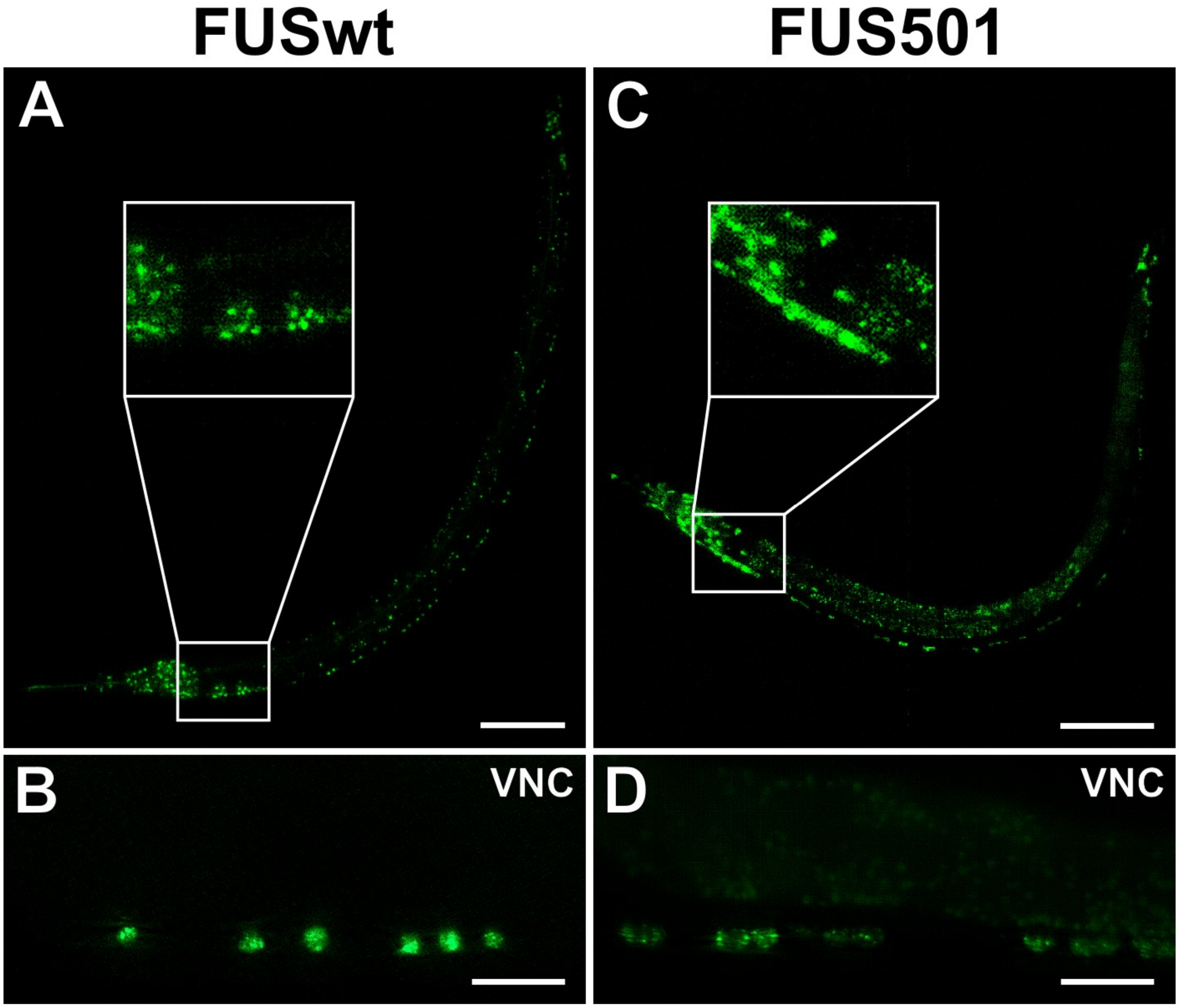

Figure S3: Structured illumination micrographs of FUSwt and FUS501 expression in living worms. FUSwt and FUS501 were imaged in living L3 larvae via their GFP-tags. Shown are maximum intensity projections of z-stacks. (A) Overview of FUSwt expression in the whole larva. Scale bar: 50 µm. (B) FUSwt expression in the ventral nerve cord (VNC). Scale bar: 10 µm. Note how FUSwt is located in distinct circular patterns, presumably within nuclei. (C) Overview of FUS501 expression in the whole larva. Scale bar: 50 µm. (D) FUS501 expression in the ventral nerve cord (VNC). Scale bar: 10 mm. Note how FUS501 is distributed more diffusely and forms small puncta, presumably aggregates.

Biology Open • Supplementary information
